# Oral health conditions and correlates: a National Oral Health Survey of Rwanda

**DOI:** 10.1080/16549716.2021.1904628

**Published:** 2021-04-26

**Authors:** Donna M. Hackley, Shruti Jain, Sarah E. Pagni, Matthew Finkelman, Joseph Ntaganira, John P. Morgan

**Affiliations:** a Department of Oral Health Policy and Epidemiology, Harvard School of Dental Medicine, Boston, USA; b Department of Public Health and Community Service, Tufts University School of Dental Medicine, Boston, USA; c Division of Biostatistics and Experimental Design, Tufts University School of Dental Medicine, Boston, USA; d School of Public Health, University of Rwanda, Kigali, Rwanda

**Keywords:** Global oral health, community-based intervention and capacity building, primary health care, quality of life, national survey and surveillance Rwanda

## Abstract

**Background**: Dental diseases are chronic, lifelong and preventable yet affect over half the world’s population. Personal oral hygiene practices and socio-economic factors contribute to oral health outcomes affecting oral health quality of life. Integrating basic oral care within community level health systems increases accessibility and availability of oral health resources.

**Objective**: National Oral Health Survey of Rwanda (NOHSR) data were investigated for associations of socio-demographic characteristics, personal oral hygiene practices, oral health outcomes, and oral health quality of life indicators.

**Methods**: Data were analyzed and descriptive statistics calculated. Multivariable logistic regression models were developed to assess associations between untreated caries, calculus, and pain with various independent variables (demographics and personal oral hygiene practices). Additional logistic regression models examined associations between quality of life indicators and the aforementioned independent variables as well as untreated caries and pain.

**Results**: Those who did not use a toothbrush (62.7%), or toothpaste (70.0%), and cleaned their teeth less than once per day (55.3%) had a higher prevalence of untreated caries. Approximately one-third of those in rural areas cleaned their teeth once per day or more compared to two-thirds of those in urban areas (35.4% vs. 71.2%). Those cleaning their teeth less than once daily were estimated to have 56.0% higher odds of caries than those who cleaned their teeth once a day or more (OR = 1.56, [95% CI 1.25–1.95]). Those with secondary education or higher and those with skilled jobs demonstrated more frequent teeth cleaning and higher toothbrush and toothpaste use. Quality-of-life indicators varied significantly with untreated caries and pain.

**Conclusion**: Socio-economic, individual, and workforce characteristics are important considerations when assessing oral health outcomes. This study investigated social demographic disparities in relation to oral health related behaviors and outcomes. This information can help guide oral health care programming in Rwanda.

## Background

Largely preventable, dental diseases are chronic and lifelong conditions [1] inextricably tied to general health and well-being [2]. Evolving research points toward evidence that periodontal disease is linked to systemic conditions such as diabetes and cardiovascular disease [3–5]. Approximately 3.5 billion people worldwide exhibit active oral disease [6]. The World Dental Federation (FDI) estimates that 60–90% of schoolchildren and nearly 100% of adults worldwide have experienced tooth decay in their lifetimes [7]. The Global Burden of Disease 2010 Study identified dental decay in permanent teeth as the most prevalent condition out of 291 reported diseases and injuries. Severe periodontitis in adults and untreated caries in deciduous teeth ranked sixth and 10th respectively [8]. While oral health promotion interventions that target individual behavior change have shown some success, oral disease prevention and management that take into account social determinants warrant a more comprehensive approach [9].

Dental biofilm or plaque is a major biologic determinant of oral diseases [10]. Untreated caries and periodontal disease are related, in part, to personal oral hygiene practices designed to remove this dental biofilm. Routinely performed oral hygiene (tooth brushing) with fluoridated toothpaste is the most important behavioral factor affecting both dental caries and periodontal disease [9]. However, behavioral preventive approaches alone do not address oral health inequalities [11]. Major non-communicable diseases (NCDs), including oral diseases, have been associated with components of socio-economic status such as education, income, and social position [9,12]. Affordability and availability are challenges that result in disparities in accessing oral health care especially in rural and disadvantaged populations [12]. Oral health services tend to be centralized in urban locations with most care rendered as palliative or emergent rather than as preventive or restorative [12].

**Figure 1.**
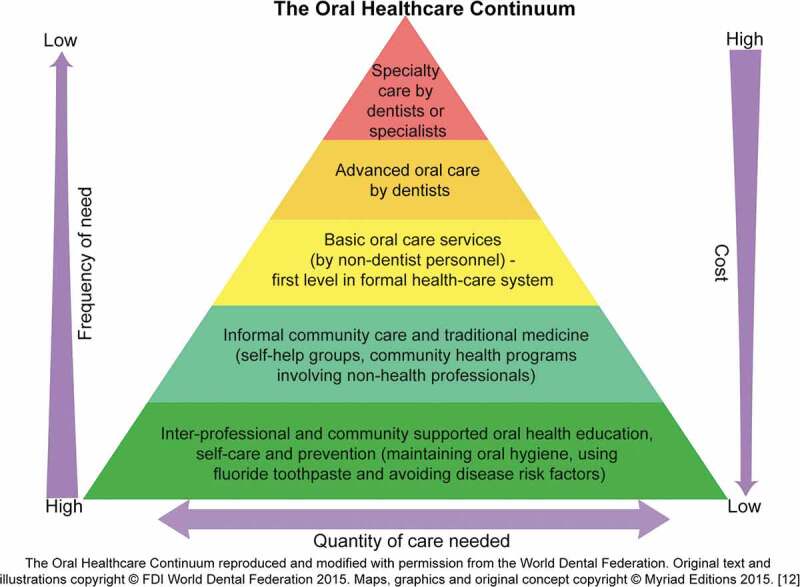
The Oral Healthcare Continuum

The Primary Health Care (PHC) model proposed in the 1978 World Health Organization’s (WHO) Declaration of Alma-Ata emphasized a shift from expensive, patient-centered, hospital-based therapeutic care to community-centered, locally accessible, cost-effective preventive care that complements oral care by oral health professionals [13]. The World Dental Federation (FDI) adapted the primary care model and graphically depicted its pyramid of primary oral healthcare known as the ‘Oral Healthcare Continuum’. (Figure 1) This model emphasizes the need for inexpensive community based oral health promotion and disease management services accessible to a large portion of the population (base of the pyramid). The inverse relationship of costs and services demonstrates cost-effective approaches by addressing frequency-of-need and cost-of-care issues in relation to a population’s need and the level of training of the workforce [12].

In 2019, ‘tooth and gum disease’ ranked as the most commonly recorded complaint at district hospitals in Rwanda [14]. A previous publication described the Rwandan context and development of the first National Oral Health Survey of Rwanda (NOHSR), socio-demographics of the study population, and oral disease burden [15]. The aim of this study was to analyze selected variables from the NOHSR dataset to provide a better understanding of oral health in Rwanda and inform strategies for oral disease prevention and management. The objectives of this study were as follows: a) report data regarding selected personal oral hygiene practices b) investigate associations of oral health outcomes with demographic variables and personal oral hygiene practices, and c) investigate quality of life with oral health outcomes and demographic variables.

## Methodology

### Data source

This manuscript was developed from results of specialized analyses of the dataset from the National Oral Health Survey Rwanda (NOHSR) conducted in 2016. The NOHSR study design was based on the World Health Organization’s (WHO) oral health basic survey methods [16]. Structured interviewer-administered questionnaires collected demographic, oral health practice, oral health behavior and quality-of-life-information. Oral epidemiologic screenings recorded oral health status. The data collection instrument was developed from questions and oral health indicators available from the WHO [16]. Data collection formats were developed for children (ages 2–11 years), adolescents (ages 12–17 years) and adults (ages 18 years and above). Each questionnaire was pretested for face and content validity and then translated into Kinyarwanda. Parents or guardians were present at the time of the survey and provided information as required for children and adolescents who were 17 years of age or younger.

Site selection and sample size were based on the WHO Oral Health Surveys Pathfinder stratified cluster methodologies in consultation with the National Institute of Statistics Rwanda (NISR). This resulted in the selection of 15 sample sites representing four provinces of Rwanda and the capital city of Kigali. Sites were randomly selected to reflect the country’s approximate urban/rural ratio (20%/80%) and provincial population distributions [17].

The study population included five age groups (2–5, 6–11, 12–19, 20–39, and 40+ years). These age groups were selected based on accepted oral health sampling domains [18]. A minimum of 25 subjects in each age group for each site was determined based on the Pathfinder methodology and pilot oral health data available in Rwanda. Individuals participating in the survey represented households randomly selected by the community health worker responsible for each site. Participants had to be a resident of the selected community.

A single survey team of seven calibrated Rwandan dental therapists collected data at each of the 15 selected sites. Each data collector was calibrated against a gold standard examiner and recalibrated at the conclusion of weeks one, two, and four of the six-week data collection period. A previous publication describes the specific details of the methodology for the NOHSR and baseline data regarding the oral disease burden in Rwanda [15].

### Analysis

For this study selected variables from the 2016 NOHSR (Table 1) were analyzed using the Statistical Package for Social Sciences (SPSS) Version 22 (IBM Corp, Armonk, NY), Stata version 13.1 (Stata Corp LP, College Station, TX) and SAS version 9.4 (SAS Institute Inc., Cary, NC). For analysis, the frequency of cleaning teeth variable (self-reported by adult and adolescent participants and parent/guardian-reported for children) was categorized as greater than or equal to once per day if the participant reported cleaning their teeth one time per day or more. Cleaning frequency was categorized as less than once per day if the participant reported cleaning her/his teeth less than one time per day or not at all. Answer options in the survey accounted for the possibility that participants reported cleaning their teeth occasionally, but less than once per day, when aggregated across days (e.g. cleaning once every two days). Data regarding education and occupation were only available for analysis of age groups 20–39 and 40+ years. Descriptive statistics (counts and percentages for categorical variables, means and standard deviations for continuous variables) were calculated. The chi-square test (Tables 2–5) was used to determine associations between categorical oral health and demographic variables; oral health quality of life indicators and variables such as demographics, pain and untreated caries; and, oral health variables with each other.

**Table 1.** Definitions of Selected Variables

**Independent Variable**	**Definition**
Sex	Male or female
Age Groups	In Years (2–5, 6–11, 12–19, 20–39, 40+)
Education	Participants (age ≥20 years) indicate their highest level of education attended (None, Any primary, Some secondary, Secondary or more)
Geographic Location	Live in urban or rural area as defined by National Institute of Statistics Rwanda
Medical Insurance	Medical coverage that pays for medical or surgical care (Yes, No)
Occupation	Participants (age ≥20 years) indicate the kind of work they mainly do (Agriculture and Unskilled manual; Skilled, Technical, Professional; Student or Not working)
**Oral Health Indicators**	**Definition**
Untreated Caries	Visually detectable cavitated carious lesion on at least one deciduous or one permanent tooth (Yes, No)
Calculus	Calcified dental plaque noted by visual inspection only (Yes, No)
Frequency of cleaning	Frequency with which participant reported cleaning his/her teeth at least once per day (Yes, No)
Pain	Felt painful aching in the mouth within the 12 months preceding the survey (Yes, No)
Treatment urgency	Presence of oral pain, infection, or swelling or any condition that is life threatening (Yes, No)
Use of toothbrush	Participant indicated using a toothbrush to clean his/her teeth (Yes, No)
Use of toothpaste	Participant indicated using toothpaste to clean his/her teeth (Yes, No)
**Quality of Life Indicators**	**Definition**
Difficulty chewing	Difficulty chewing in the past 12 months because of problems with teeth, mouth or dentures (Yes, No)
Difficulty working	Difficulty doing usual jobs, attending school or participating in social activities in the past 12 months because of problems with teeth, mouth or dentures (Yes, No)
Feel self-conscious	Felt self-conscious or embarrassed within the preceding year because of teeth, mouth or dentures (Yes, No)

**Table 2.** Oral health indicators by demographic indicators

		Oral health indicators					
Demographic Characteristics		Untreated Caries			Calculus		
		Yes (n) %	No (n) %	p-value ^§^	Yes (n) %	No (n) %	p-value ^§^
Total		1138 (54.3)	959 (45.7)	NA	623 (29.7)	1474 (30.3)	NA
Sex	Male	439 (53.8)	377 (46.2)	.73	226 (27.7)	590 (72.3)	.11
	Female	699 (54.6)	582 (45.4)		397 (31.0)	884 (69.0)	
Age (Years)	2–5	199 (49.8)	201 (50.3)	<.0001	0 (0.0)	400 (100.0)	<.0001
	6–11	239 (56.2)	186 (43.8		23 (5.4)	402 (94.6)	
	12–19	177 (43.1)	234 (56.9)		83 (20.2)	328 (79.8)	
	20–39	250 (56.2)	195 (43.8)		227 (51.0)	218 (49.0)	
	40+	273 (65.6)	143 (34.4)		290 (69.7)	126 (30.3)	
Geographic Location	Urban	271 (49.3)	279 (50.7)	.006	137 (24.9)	413 (75.1)	.004
	Rural	867 (56.0)	680 (44.0)		486 (31.4)	1061 (68.6)	
Medical Insurance*	Yes	928 (54.0)	789 (46.0)	.73	504 (29.4)	1213 (70.7)	.47
	No	208 (55.0)	170 (45.0)		118 (31.2)	260 (68.8)	
Education**	None	173 (65.5)	91 (34.4)	.18	191 (72.4)	73 (27.7)	<.0001
	Any primary	287 (59.5)	195 (40.5)		271 (56.2)	211 (43.8)	
	Some secondary	34 (53.1)	30 (46.9)		33 (51.6)	31 (48.4)	
	Secondary or more	28 (56.0)	22 (44.0)		21 (42.0)	29 (58.0)	
Occupation**	Agriculture and Unskilled	400 (61.0)	256 (39.0)	.86	410 (62.5)	246 (37.5)	.002
	Skilled, Technical and Professional	72 (58.5)	51 (41.5)		56 (45.5)	67 (54.5)	
	Not working and Student	50 (61.7)	31 (38.3)		50 (61.7)	31 (38.3)	

^§^*All p values derived from χ2 tests.*

*Total n = 2097. Missing data represents less than 1%.

**Data reported for participants ≥20 years of age (n = 860).

**Table 3.** Personal oral hygiene indicators by demographic characteristics

		Personal Oral Hygiene Practices								
Demographic Characteristics		Frequency of cleaning^±^			Use of toothbrush			Use of toothpaste		
		≥1x/d	<1x/d	p-value ^§^	Yes	No	p-value ^§^	Yes	No	p-value ^§^
Characteristic		n (%)	n (%)		n (%)	n (%)		n (%)	n (%)	
Total		938 (44.7)	1159 (55.3)	NA	781 (37.2)	1315 (62.7)	NA	671 (32.0)	1423 (70.0)	NA
Sex*	Male	327 (40.2)	487 (59.8)	<.0001	286 (35.1)	529 (64.0)	.09	252 (30.9)	563 (69.1)	.39
	Female	611 (47.7)	669 (52.3)		495 (38.6)	786 (61.4)		419 (32.8)	860 (67.2)	
Age* (Years)	2-5	83 (20.8)	316 (79.2)	<.0001	69 (17.3)	331 (82.8)	<.0001	56 (14.0)	343 (86.0)	<.0001
	6-11	137 (32.3)	287 (67.7)		106 (24.9)	319 (75.1)		95 (22.4)	330 (77.6)	
	12-19	219 (53.3)	192 (46.7)		178 (43.3)	233 (56.7)		151 (36.8)	259 (63.2)	
	20-39	288 (64.8)	156 (35.1)		260 (58.4)	185 (41.6)		222 (50.0)	222 (50.0)	
	40+	211 (50.7)	205 (49.3)		168 (40.5)	247 (59.5)		147 (35.3)	269 (64.7)	
Geo. Loc.*	Urban	391 (71.1)	159 (28.9)	<.0001	391 (71.2)	158 (28.8)	<.0001	365 (66.4)	185 (33.6)	<.0001
	Rural	547 (35.4)	1000 (64.6)		390 (25.2)	1157 (74.8)		306 (19.8)	1238 (80.2)	
Med.Ins.*	Yes	766 (44.6)	951 (55.4)	.75	656 (38.2)	1060 (61.8)	.06	567 (33.1)	1147 (66.9)	.04
	No	172 (45.5)	206 (54.5)		125 (33.1)	253 (66.9)		104 (27.5)	274 (72.5)	
Ed.**	None	111 (42.1)	153 (58.0)	<.0001	67 (25.4)	197 (74.6)	<.0001	53 (20.1)	211 (79.9)	<.0001
	Any primary	292 (60.6)	190 (39.4)		263 (54.6)	219 (45.4)		224 (46.6)	257 (53.4)	
	Some secondary	55 (85.9)	9 (14.1)		54 (84.4)	10 (15.6)		50 (78.1)	14 (21.9)	
	Secondary or more	41 (82.0)	9 (18.0)		44 (89.8)	5 (10.2)		42 (84.0)	8 (16.0)	
Occ.**	Agriculture and Unskilled	326 (49.7)	330 (50.3)	<.0001	262 (39.9)	394 (60.1)	<.0001	215 (32.8)	440 (67.2)	<.0001
	Skilled, Technical and Professional	112 (91.1)	11 (8.9)		111 (91.0)	11 (9.0)		105 (85.4)	18 (14.6)	
	Not working and Student	61 (75.3)	20 (24.7)		55 (67.9)	26 (32.1)		49 (60.5)	32 (39.5)	

^±^Frequency of cleaning ≥1x/d = participant reported cleaning their teeth one time per day or more; < 1x/d = participant reported cleaning their teeth less than one time per ^§^*All p values derived from χ2 tests.* *Total n=2097. Missing data represents less than 1% (Geo. Loc. = Geographic Location; Med. Ins. = Medical Insurance). **Data reported for participants ≥20 years of age (n=860). (Ed. = Education; Occ. = Occupation). day or not at all.

**Table 4.** Quality of life indicators by oral health outcomes and selected demographic characteristics

		Quality of Life Indicators								
Oral Health Outcomes and Selected Demographic Characteristics		Difficulty Working^+^			Difficulty Chewing			Feel Self Conscious		
		Yes n (%)	No n (%)	p-value ^§^	Yes n (%)	No n (%)	p-value ^§^	Yes n (%)	No n (%)	p-value ^§^
Total		740 (35.4)	1353 (64.6)	NA	882 (42.2)	1206 (57.8)	NA	755 (36.2)	1330 (63.8)	NA
Pain*	Yes	699 (52.2)	639 (47.8)	<.0001	831 (62.3)	504 (37.8)	<.0001	715 (53.7)	616 (46.3)	<.0001
	No	41 (5.4)	714 (94.6)		51 (6.8)	702 (93.2)		40 (5.3)	714 (94.7)	
Untreated Caries*	Yes	537 (47.3)	598 (52.7)	<.0001	625 (55.3)	505 (44.7)	<.0001	555 (49.1)	576 (50.9)	<.0001
	No	203 (21.2)	755 (78.8)		257 (26.8)	701 (73.2)		200 (21.0)	754 (79.0)	
Location*	Urban	144 (26.2)	406 (73.8)	<.0001	179 (32.7)	369 (67.3)	<.0001	159 (29.0)	390 (71.0)	<.0001
	Rural	596 (38.6)	947 (61.4)		703 (45.7)	837 (54.4)		596 (38.8)	940 (61.2)	
Medical Insurance*	Yes	593 (34.6)	1120 (65.4)	.12	706 (31.3)	1003 (58.7)	.07	606 (35.5)	1103 (64.5)	.11
	No	147 (38.9)	231 (61.1)		175 (46.4)	202 (53.6)		149 (39.8)	225 (60.2)	
Education** (≥ 20 years)	None	175 (66.5)	88 (33.5)	.02	189 (72.1)	73 (27.9)	.04	173 (65.8)	90 (34.2)	.01
	Any primary	285 (59.3)	196 (40.8)		319 (66.3)	162 (33.7)		281 (58.7)	198 (41.3)	
	Some secondary	30 (46.9)	34 (53.1)		36 (56.3)	28 (43.8)		28 (43.8)	36 (56.3)	
	Secondary or more	27 (54.0)	23 (46.0)		29 (58.0)	21 (42.0)		26 (52.0)	24 (48.0)	
Occupation** (≥ 20 years)	Agriculture and Unskilled	414 (63.3)	240 (36.7)	.0003	448 (68.6)	205 (31.4)	.11	403 (61.7)	250 (38.3)	.02
	Skilled, Technical and Professional	70 (56.9)	53 (43.1)		78 (63.4)	45 (36.6)		68 (55.3)	55 (44.7)	
	Not working and Student	33 (40.7)	48 (59.3)		47 (58.0)	34 (42.0)		37 (46.3)	43 (53.8)	

+Difficulty doing usual jobs, attending school or participating in social activities. ^§^*All p values derived from χ2 tests.* *Total n=2097. Missing data represents less than 1%. **Data reported for participants ≥20 years of age (n=860).

**Table 5.** Oral health outcomes by personal oral hygiene practices and pain

		Oral Health Outcomes					
Personal Oral Hygiene Practices and Pain		Untreated Caries			Calculus		
		Yes n (%)	No n (%)	p-value ^§^	Yes n (%)	No n (%)	p-value ^§^
Total*		1137 (54.2)	959 (45.8)	NA	622 (29.7)	1474 (60.3)	NA
Use of Toothbrush*	Yes	379 (48.5)	402 (51.5)	<.0001	238 (30.5)	543 (69.5)	.54
	No	758 (57.6)	557 (42.4)		384 (29.2)	931 (70.8)	
Use of Toothpaste*	Yes	333 (49.6)	338 (50.4)	.004	212 (31.6)	459 (68.4)	.18
	No	803 (56.4)	620 (43.6)		409 (28.7)	1014 (71.3)	
Frequency of cleaning*	≥ 1x/day^±^	451 (48.1)	487 (51.9)	<.0001	311 (33.2)	627 (66.8)	.002
	<1x/day	687 (59.3)	472 (40.7)		312 (26.9)	847 (73.1)	
Pain	Yes	865 (64.6)	475 (35.4)	<.0001	513 (38.3)	827 (61.7)	<.0001
	No	273 (36.1)	484 (63.9)		110 (14.5)	647 (85.5)	

^§^*All p values derived from χ2 tests.*

*Total n = 2097. Missing data represents less than 1%.

(±) Frequency of cleaning ≥1x/d = participant reported cleaning their teeth one time per day or more; < 1x/d = participant reported cleaning their teeth less than one time per day or not at all.

Multivariable logistic regression models (Table 6) were developed and tested to assess associations adjusted for potential confounding. In particular, models for the dependent binary (yes/no) variables of untreated caries, calculus and pain were constructed with the following covariates (all of which were considered as confounders): sex, age, location, medical insurance, cleaning teeth less than once per day, and toothbrush use. Further models were constructed for the dependent binary variables of difficulty working, difficulty chewing and self-consciousness with each model initially including all of the covariates mentioned above as well as untreated caries and pain as further covariates - all of which were considered as confounders. However, medical insurance was ultimately excluded from the model for untreated caries due to lack of fit (based on the Hosmer-Lemeshow test for goodness of fit which was also used to confirm the fit of each model).

**Table 6.** Binary logistic regression models*^§.^*

Characteristics	Oral Health Outcomes		
	Untreated Caries n = 2094	Calculus n = 2094	Pain n = 2094
Demographics	OR [95% CI]	OR [95% CI]	OR [95% CI]
Sex			
Male	1.03 [0.86–1.24]	1.89 [1.46–2.45]*	0.94 [0.77–1.15]
Female	1.00	1.00	1.00
Age (Years)			
2–5	0.43 [0.32–0.58]*	<.001 [<.001–0.01]*	0.09 [0.06–0.13]*
6–11	0.60 [0.45–0.80]*	0.02 [0.01–0.03]*	0.18 [0.13–0.25]*
12–19	0.40 [0.30–0.53]*	0.09 [0.07–0.13]*	0.22 [0.15–0.31]*
20–39	0.74 [0.56–0.98]*	0.50 [0.37–0.67]*	0.42 [0.29–0.61]*
40+	1.00	1.00	1.00
Geographic Location			
Urban	0.95 [0.76–1.19]	0.82 [0.61–1.11]	0.77 [0.60–0.98]*
Rural	1.00	1.00	1.00
Medical Insurance			
Yes	-**	1.23 [0.91–1.67]	1.17 [0.91–1.51]
No	-**	1.00	1.00
Personal Oral Hygiene Practices			
Cleaning Freq. < 1^±^	1.56 [1.25–1.95]*	1.12 [0.84–1.49]	0.87 [0.68–1.11]
Cleaning Freq. ≥ 1	1.00	1.00	1.00
No use of Toothbrush	1.20 [0.94–1.52]	2.01 [1.48–2.72]*	0.74 [0.57–0.98]*
Use of Toothbrush	1.00	1.00	1.00
Characteristics	Quality of Life Indicators		
Oral health Outcomes	Difficulty Working n = 2090	Difficulty Chewing n = 2085	Feel Self-conscious n = 2082
No Untreated Caries	0.40 [0.31–0.51]*	0.40 [0.32–0.51]*	0.34 [0.28–0.46]*
Untreated Caries	1.00	1.00	1.00
No Pain	0.08 [0.06–0.12]*	0.07 [0.05–0.09]*	0.08 [0.06–0.11]*
Pain	1.00	1.00	1.00
Geographic Location			
Urban	0.44 [0.32–0.59]*	0.41 [0.31–0.56]*	0.53 [0.40–0.71]*
Rural	1.00	1.00	1.00
Medical Insurance			
Yes	1.34 [0.99–1.79]	1.40 [1.04–1.88]*	1.33 [0.99–1.77]
No	1.00	1.00	1.00
Sex			
Male	0.79 [0.62–1.01]	0.86 [0.68–1.10]	0.84 [0.67–1.07]
Female	1.00	1.00	1.00
Age (Years)			
2–5	0.10 [0.07–0.16]*	0.14 [0.09–0.21]*	0.14 [0.10–0.21]*
6–11	0.16 [0.11–0.22]*	0.15 [0.10–0.21]*	0.16 [0.11–0.22]*
12–19	0.19 [0.13–0.27]*	0.19 [0.13–0.27]*	0.22 [0.15–0.31]*
20–39	0.73 [0.53–1.01]	0.54 [0.38–0.78]*	0.55 [0.40–0.75]*
40+	1.00	1.00	1.00
Personal Oral Hygiene Practices			
Cleaning Freq. < 1^±^	2.14 [1.60–2.87]*	1.44 [1.08–1.93]	1.25 [0.94–1.66]
Cleaning Freq. ≥ 1	1.00	1.00	1.00
No use of Toothbrush	0.50 [0.37–0.69]*	0.62 [0.45–0.84]*	0.70 [0.50–0.95]*
Use of Toothbrush	1.00	1.00	1.00

^§^All results derived from multivariable logistic regression. All odds ratios presented are adjusted odds ratios; for each outcome in the table, the multivariable logistic regression model included all covariates listed underneath that outcome.

*Statistically significant (p < .05).

**Medical Insurance was not included in the multivariable logistic regression model for untreated caries.

^±^*Frequency of cleaning ≥1x/d = participant reported cleaning their teeth one time per day or more; < 1x/d = participant reported cleaning their teeth less than one time per day or not at all.*

The assumption of lack of multicollinearity was assessed using Variance Inflation Factor (VIF) values. From previous research [19], a VIF of 2.5 or higher was considered to indicate substantial collinearity. Based on this criterion, toothbrush and toothpaste use were the only covariates to exhibit substantial collinearity, due to their high level of correlation. Since toothbrush use was of greater interest as a covariate, it was retained in each final model, whereas toothpaste use was excluded from the models. Adjusted odds ratios and corresponding 95% confidence intervals were calculated based on the final logistic regression models. McFadden pseudo R^2^ values were also calculated.

## Results

As reported by the NOHSR, the study population consisted of 2097 participants (61.1% female), mean (SD) age of 22.5 (19.6) years (range 2–104 years). Demographic information of the study group including level of education, rurality, occupation, and participation in community-based health insurance (CBHI) reflected the population of Rwanda. Approximately three quarters (73.8%) lived in rural communities. For participants ages 20 years and above, the majority (86.8%) had completed primary school or lower grade level. Over three quarters of the participants (78.7%) had medical insurance coverage. Consistent with a high disease burden, over half of the study sample (54.3%) had untreated caries with a significantly higher prevalence in older age groups and rural areas. Nearly two-thirds (63.9%) had experienced painful aching in the mouth within the past 12 months. For those ages 20 years and above, over 50% of the participants reported that oral health problems were affecting their quality of life [15].

Further analysis of the NOHSR dataset for this study showed that most people 20 years of age and older (76.3%) reported their occupation as agriculture or unskilled labor. Selected variables of untreated caries and calculus varied significantly with age and geographic location (p < .05) but not by sex or medical insurance (p > .05). The highest prevalence of caries (65.6%) and calculus (69.7%) was reported in those 40 years of age and older. Caries prevalence was higher among those living in rural areas verses urban areas (56.0% vs. 49.3%). The reported presence of calculus was also higher in rural areas compared to urban areas (31.4% vs. 24.9%). For those 20 years of age and older, the presence of calculus varied significantly with level of education and occupation (p < .05). Less calculus was present in individual with higher levels of education and those engaged in skilled labor. Untreated caries did not vary significantly with education or occupation (p > .05). (Table 2)

Cleaning frequency varied significantly by sex, age, geographic location, level of education and occupation (p < .05). Overall 55.3% of the study population cleaned their teeth less than once per day or not at all. The frequency of cleaning teeth once per day or more was higher among females than males (47.7% vs. 40.2%) and those living in urban areas versus rural areas (71.1% vs. 35.4%). Cleaning frequency was highest for participants 20–39 years of age (64.8%) and lowest for those ages 2–5 years (20.8%). For the study population 20 years of age or older, the frequency of cleaning once per day or more was greater for people with higher levels of education and those engaged in skilled, technical or professional occupations. Frequency of cleaning did not vary significantly based on having medical insurance (p > .05). (Table 3)

Of the study population, 62.7% reported not using a toothbrush and 70.0% reported not using toothpaste. Use of a toothbrush and toothpaste varied significantly by age, geographic location, education and occupation (p < .05), but not by sex (p > .05). Toothbrush use was highest for participants 20–39 years of age (58.4%) and lowest for those ages 2–5 years (17.3%). Participants living in urban areas reported more toothbrush use than those living in rural areas (71.2% vs. 25.2%). For participants 20 years and older, toothbrush and toothpaste use was reported more often by those with higher levels of education and more skilled labor. The use of toothpaste was significantly higher among participants with medical insurance (p < .05). (Table 3) Approximately 83.1% (648/780) of those using a toothbrush reported using toothpaste as well. Of those not using a toothbrush approximately 1.7% (22/1313) reported using toothpaste.

Indicators for oral health quality of life varied significantly with oral health outcomes and selected demographic characteristics. Study participants without pain or untreated caries reported less difficulty working, less difficulty chewing, and less feelings of self-consciousness (p < .05). Difficulty working and feelings of self-consciousness varied significantly with geographic location, level of education and type of occupation (p < .05). Participants living in urban areas reported less difficulty working and feeling less self-conscious due to oral problems. Those 20 years of age and older with higher levels education also reported less difficulty working and feeing less self-conscious about their oral conditions (p < .05). None of the quality-of life indicators varied based on medical insurance (p > .05). (Table 4)

In bivariate analyses, toothbrush use, toothpaste use, frequency of cleaning and oral pain varied significantly with untreated caries (p < .05). Participants who did not use a toothbrush, did not use toothpaste, and cleaned their teeth less than one time per day or not at all had a higher prevalence of untreated caries (57.6%, 56.4% and 59.3% respectively). Nearly two-thirds (64.6%) of those with oral pain within the last year had untreated caries. The presence of calculus did not vary significantly with the use of toothbrush or toothpaste (p > .05). (Table 5)

After excluding toothpaste use from all models and excluding medical insurance from the model for untreated caries (as described in the Analysis section), all assumptions for multivariable logistic regression were met in the final models. Table 6 shows the adjusted odds ratios and corresponding 95% confidence intervals for each multivariable logistic regression model. Within the table, all variables shown for a given model were included as covariates (potential confounders) in that model. After adjusting for potential confounders via the multivariable logistic regression models, participants not cleaning their teeth at least once per day were estimated as having 56.0% higher odds for untreated caries than those who cleaned once a day or more. Furthermore, in the multivariable logistic regression model, statistically significant findings indicated that participants who did not use a toothbrush were estimated at 101.0% higher odds of having calculus than those who used a toothbrush. Those living in urban areas were estimated to have 23.0% lower odds of having pain than those in rural areas. (Table 6) The pseudo R ^2^ was 0.052 for the untreated caries model, 0.495 for the calculus model and 0.190 for the pain model.

Quality-of-life predictors in the multivariable logistic regression models included untreated caries, pain, location, medical insurance, age, frequency of cleaning teeth, and use of a toothbrush. Participants without untreated caries were estimated to have 60.0% lower odds of having difficulty working than those with untreated caries. Those without pain in their mouths were estimated to have 92.0% lower odds of having difficulty working than those who had pain. Predictors for the two additional quality of life indicators, difficulty chewing and self-consciousness, followed a similar pattern for untreated caries and pain. Participants living in urban areas had lower odds of adverse impacts on oral health quality of life than those living in rural areas. A cleaning frequency of less than once per day resulted in 114.0% higher odds of difficulty working than if cleaning was done more frequently. However, not using a toothbrush did not have higher odds for difficulty working, difficulty chewing, or feeling of self-consciousness. Younger age groups had lower odds for the quality of life indicators consistent with age related oral disease. (Table 6) Pseudo R ^2^ values were 0.488 for difficulty working, 0.515 for difficulty chewing, and 0.469 for feeling self-conscious.

## Discussion

The data provided by the NOHSR afforded the opportunity to assess factors related to oral disease. Building on the first manuscript describing the NOHSR [15], we further analyzed the NOHSR dataset to investigate key outcome and descriptive variables to better understand the oral health of the surveyed population. Specifically, we investigated oral health indicators in relation to demographic information, personal oral hygiene practices, and oral health quality of life indicators. This provided further insight into the substantial oral disease burden in Rwanda and the impact of selected determinants in relation to the reported oral health outcomes.

Personal oral hygiene practices such as the use of a toothbrush with fluoridated toothpaste, professional cleaning, oral hygiene instruction and motivation, dietary advice, and professional fluoride application contribute to the management of dental caries and periodontal disease [9,20–24]. The NOHSR identified that 70.6% of the study population never visited an oral health provider and of those who did, 98.7% sought care for pain relief [15]. Given the oral healthcare system was predominantly used for pain relief, the focus of professional visits on pain management diminishes the opportunity for beneficial contributions of professional services such as cleaning, oral health promotion, prevention, and oral disease management services. Our current findings are consistent with an identified need for the development of strategies that improve personal oral hygiene practices including the frequency of tooth cleaning and the use of fluoridated toothpaste. Specific age groups, those living in rural areas, those with lower education levels, those in less skilled occupations and students were specifically challenged in regard to their self-performed oral hygiene.

Oral diseases impact well-being and quality of life [25–27]. Oral discomfort and pain can affect chewing, talking and social roles [25]. Bivariate analysis showed that the adverse impact on quality of life was greater when untreated dental caries or pain were reported. Those in rural locations, with less than secondary education, and who engaged in agricultural and unskilled occupations reported the highest percentage of adverse impact on oral health quality of life. Although other studies do not report a direct correlation between the extent of carious lesions and whether pain and discomfort are felt [20], the dataset showed nearly two-thirds of the study participants with oral pain had untreated caries.

The lack of available data limits the understanding of oral health behavior and its impact on oral health outcomes as well as its overall effect on health [28]. Logistic regression models in our study provided additional insights into the complexity of oral diseases. The results of the logistic regression model for oral-health-quality of life outcomes reinforce that the factors of age, untreated caries, pain, geographic location and self-performed oral hygiene practices are important considerations when contextualizing oral health in Rwanda.

Access to adequate oral healthcare is an important factor in oral health outcomes and is a challenge faced by one-third of the global population [12]. Even health systems with favorable oral health workforce provider-to-patient ratios approaching 1:2000 [9], often fail to meet existing oral health treatment and disease prevention needs [1,6,29]. In developing nations such as Rwanda with small workforce-to-population ratios estimated in 2015 at 1:90,000 [14,30], overcoming oral health disparities presents substantial challenges. Nearly all Rwandans who sought oral health treatment at district hospitals were seeking pain relief [15], requiring the limited oral health workforce efforts at those facilities to be focused on pain relieving therapies.

Approaches to oral disease management that incorporate interventions targeted at individual health behaviors as well as at the broader community and societal levels are consistent with current socio-ecological frameworks that recognize the interplay among multi-level factors influencing oral health outcomes [1,11,31]. The Oral Healthcare Continuum (OHC) [12] offers an integrative framework leveraging community health workforce efforts to deliver community-accessible cost-effective oral health promotion and disease prevention services. Simultaneously the OHC recognizes the need for professionally trained oral health providers [12]. The Basic Package of Oral Care (BPOC), the only WHO-approved model for the management of oral diseases, focuses on oral urgent treatment, affordable fluoride toothpaste and atraumatic restorative treatment. The guiding principles of BPOC center on equity and access, cost-effectiveness, community contextualization, and multi-sectorial integration [32]. Integrating models such as the BPOC within existing community-level health systems increases the accessibility and availability of oral health promotion, prevention, as well as disease management services. Systematically developing multi-level approaches that target complex demographic, personal oral health, workforce, and socio-economic determinants offers an ecologically comprehensive approach to oral disease prevention [33]. Our analysis of the NOHSR dataset provides a resource to inform the implementation of strategies including the BPOC through the OHC model to help reduce the oral disease burden in Rwanda.

Variations in study design limit the ability to compare our findings with oral health studies of East African nations. The use of oral examination verses self-reported oral health status, physical conditions of the clinical evaluations, demographic and clinical variables, study population size, and inclusion criteria vary greatly for oral health studies reported in the literature. The NOHSR used clinical evaluation methods to document oral disease prevalence. Nonetheless, studies using self-rated oral health status and outcomes in Kenya and Tanzania reported oral health outcomes in relation to selected variables of age, education, rurality, personal oral hygiene practices and oral health quality of life findings consistent with ours [34,35].

### Discussion of statistical considerations

Important to note in our analysis, while the Hosmer-Lemeshow test did not indicate a significant misfit in any of the reported models, the pseudo R^2^ values indicated the varied abilities of the models to predict the outcome. In particular, the pseudo R^2^ values ranged from 0.469 to 0.515 for the models predicting calculus, difficulty working, difficulty chewing and feeling self-conscious, whereas the values for untreated caries and pain were lower (0.052 and 0.190, respectively). Therefore, the independent variables in the models for untreated caries and pain showed limited value in predicting these outcomes.

The use of survey weights in a given data analysis is also a statistical consideration. Gelman [36] preferred unweighted regression models in order to avoid the disadvantages associated with such weights. His recommendations were similarly incorporated in our study. Previous research using the WHO Oral Health Surveys Pathfinder methodology also employed unweighted analyses [37,38]. Comparison of the different statistical procedures when using the Pathfinder method in oral health research would be an interesting topic for future work.

### Strengths and limitations

The Pathfinder methodology is advocated by the WHO for the planning of oral care services [16]. There are certain methodological considerations. Participant recruitment continued until cluster quotas were reached, leading to possible volunteer bias. No information was known about non-participants in the study, limiting conclusions regarding selection bias. The survey relied, in part, on self-reported information that could lead to recall bias with over reporting or underreporting due to social desirability. Untreated caries and calculus were assessed by visual exam only and therefore, the true burden of diseases was underrepresented. Information on education and occupation was consistently available only for those in age groups 20–39 and 40+ years. Assessing barriers to the use of toothbrush, use of toothpaste, and frequency of cleaning was beyond the scope of the NOHSR. Despite these limitations, strengths of the study include the large sample size with representative national sampling in regard to age and randomization of site selection. Data collected from the study participants were consistent with national data reported for education, occupation and medical insurance. Pre-stratification by age and sex reduced selection bias. The cluster sampling approach ensured that the study population’s urban/rural and provincial distribution reflected that of the population of Rwanda. The NOSHR data set offers valuable insights to the complex issues regarding oral disease burden in Rwanda [15].

Future studies focused on investigating socio-economic, environmental, and individual barriers to achieving optimal oral health outcomes would assist in providing more information for developing contextually relevant oral health promotion, prevention and disease management strategies. Information regarding knowledge and attitudes toward personal oral hygiene practices and barriers to those practices is not currently available. Detailed information regarding utilization of professional oral health services as well the availability and cost of toothbrushes and fluoridated toothpaste would also be helpful to better inform oral health programming in Rwanda.

## Conclusion

Socio-economic, personal oral hygiene practices, and oral workforce characteristics are important considerations when assessing oral health outcomes. This study investigated social demographic disparities in relation to oral health related behaviors and outcomes. This information can help guide oral health-care programming in Rwanda.

## PAPER CONTEXT

Heavy disease burden and disparities, coupled with a limited workforce require innovative strategies to improve oral health. Community-based oral health promotion and disease prevention/management approaches can complement an existing, overburdened, treatment-focused workforce.
